# Non-Leachable Hydrophilic Additives for Amphiphilic Coatings

**DOI:** 10.3390/polym10040445

**Published:** 2018-04-16

**Authors:** Guillaume Gillet, Fabrice Azemar, Fabienne Faÿ, Karine Réhel, Isabelle Linossier

**Affiliations:** University of Southern Brittany, EA 3884, Laboratoire de Biotechnologie et Chimie Marines (LBCM), Institut Universitaire Européen de la Mer IUEM, F-56100 Lorient, France; fabrice.azemar@univ-ubs.fr (F.A.); fabienne.fay@univ-ubs.fr (F.F.); karine.rehel@univ-ubs.fr (K.R.); isabelle.linossier@univ-ubs.fr (I.L.)

**Keywords:** poly(ethylene glycol) (PEG), hydrophilic, non-releasable, polydimethylsiloxane, coatings, cross-linking, surface, amphiphilic, anti-bioadhesion

## Abstract

Amphiphilic surfaces are particularly effective at inhibiting the adhesion of microorganisms (bacteria, cells, microalgae, etc.) in liquid media. The aim of this study is to determine the best hydrophilic linker to promote bonding between poly(ethylene glycol) (PEG) as a hydrophilic additive and poly(dimethyl siloxane) (PDMS) as the hydrophobic matrix. Various parameters have been studied (molecular weight, linker type, and polymer end-group), as well as the efficiency of the linking, the capacity of PEG to access to the surface of the film, and overall film homogeneity. According to the results, a PDMS linker paired with a PEG moiety allows for compatibilization of the compounds during cross-linking. This compatibilization seems to provide a good bonding with the matrix and a good surface access to the hydrophilic moiety. Therefore, this structure comprising a linking function attached to the PDMS–PEG copolymer has high potential as a non-releasable additive for amphiphilic coating applications.

## 1. Introduction

Silicone elastomers such as poly(dimethyl siloxane) (PDMS) have received great attention regarding their potential use as anti-bioadhesion coatings [1]. PDMS is a soft polymer with attractive chemical and physical properties such as biocompatibility, low toxicity, flexible surface chemistry, low permeability to water, elastomeric properties, ease of fabrication and use, as well as low manufacturing costs [2]. It is widely used to reduce or prevent bioadhesion in biomedical field [3,4,5,6,7,8]. However, these kinds of coatings are prone to protein adsorption and accumulation of strongly adherent diatom slimes [9]. The addition of oil (e.g., amphiphilic copolymers) is a common technique for enhancing the biofouling-resistance properties of silicone-matrix fouling-release coatings [10]. Kavanagh et al. [11] have used two PDMS-based fluids that significantly reduced the adhesion strength of barnacles compared to unmodified elastomers. It has been reported that these “oils” are usually block copolymers that can migrate through the coatings to function as surfactants and produce a weak boundary layer affecting the surface properties [12,13]. Poly(ethylene glycol) (PEG) has been widely used as an additive for fouling release [14,15,16]. Camos Noger et al. [17] studied the diffusion ability of PEG-based amphiphilic compounds containing different hydrophobic components through a PDMS matrix via contact angle measurements. Wu et al. modified a PDMS surface with a PEG-based block copolymer to improve the anti-protein effect with respect to fluorescent tagged antibody adhesion [18]. The lubricity of PDMS surfaces has also been increased by using poly(acrylic acid)- and PEG-based block copolymers, as shown by Røn et al. [19]. Sundaram et al. [9] used the combination of PDMS and PEG chains to test the advantages over conventional silicones against diatom fouling [17,20]. Silicone oils have been classified as hazardous substances and cannot be released into water environments. While no hazards are substantiated for silicone resins, PDMS fluids are a matter of concern. Their inert oily nature and relative extreme persistence are a potential threat to aqueous environments as well as the human body. According to Nendza et al. [21], a favorable option may be the use of silicone-based coatings formulated without leaching silicone oils. This option may be achieved by using compounds with an ability to chemically bind to the network during cross-linking [22]. 

The aim of this study is to determine the optimal method of incorporating sustainable hydrophilic compounds into silicone coatings to obtain one with amphiphilic surface properties. The ability of the hydrophilic compound to remain in the coating and to access the surface presents two advantages: the durability of amphiphilic properties is increased, and the release of polymers into the environment or the body is avoided.

In this study, a hydrophilic PEG-based component was introduced into a PDMS matrix. The PDMS used contains methoxysilane end groups, which, in the presence of water and catalyst, can cross-link and form a tridimensional network. In this network, different sizes of PEG have been incorporated in an un-cross-linked blend that (1) is able to react with the cross-linking group (methoxysilane) of the PDMS network, (2) contains “unreactive” groups with respect to methoxysilane as a negative reference, and (3) contains a linker (PDMS, alkyl chain C_14_, or alkyl chain C_8_) to be used as a compatibilizer. The un-cross-linked blend was applied to the surface of polycarbonate and glass to proceed with different tests after curing. The cross-linking potential was then evaluated via soxhlet extraction and a swelling test. The surface accessibility potential of the hydrophilic compounds was characterized by contact angle measurements. Lastly, the surface homogeneity was observed by scanning electron microscopy. 

## 2. Materials and Methods

### 2.1. Materials

Poly(ethylene glycol) methyl ether (*M*_n_: 2000 g/mol, **PEG_45_**), pentenoic acid 97%, *N*,*N*′-dicyclohexylcarbodiimide 99%, 4-dimethylaminopyridine >99%, tetraethyl orthosilicate reagent grade 98%, azobisisobutyronitrile 98%, and phosphoric acid 85% in water were acquired from Sigma-Aldrich (Saint-Louis, MO, USA). Hydroxyl-terminated poly(ethylene glycol) (*M*_n_: 400 g/mol, **PEG_9_**) was provided by Biochemica (Billingham, UK). Tegomer^®^ (Essen, Germany) H-Si 2315 polydimethylsiloxane bis(hydroxyl) (*M*_n_: 2315 g/mol per manufacturer’s specifications, **PDMS_28_**) was kindly supplied by EVONIK (Essen, Germany). (3-Mercaptopropyl)trimethoxysilane > 96% was purchased from TCI (Tokyo, Japan). **PDMS-SiOH** (copolymer PEG-PDMS, mono Si–OH end function, which may react with cross-linking function, pendant chain PEG per manufacturer specifications) and 3-(triethoxysilyl)propyl isocyanate 98% are commercially available (Momentive, Waterford, NY, USA) and were used in bulk reaction (1/1.05 equiv, N_2_, 20 °C) to form **PDMS-TMS**, which was ended with bis(trimethoxysilane) and could react with cross-linking functions Trimethoxysilane-terminated poly(ethylene glycol) (*M*_n_: 350 g/mol, **PEG_6_**) was also purchased from Momentive. US-CF 2403 methyl siloxane resin with methoxy function was provided by Dow Corning (Midland, MI, USA) (*M*_w_ < 1000g/mol per manufacturer specifications) and was named **PDMS_13_**. All products were used as received.

### 2.2. Synthesis and Coating

#### 2.2.1. Synthesis of Bis(Trimethoxysilane)-Terminated Poly(Dimethyl Siloxane) (PDMS_28_) 

**PDMS_28_** was prepared via Steglich esterification followed by a thiol-ene reaction. Firstly, dihydroxyalkyl poly(dimethyl siloxane) (15 g, 1 equiv), pentenoic acid (1.94 g, 3 equiv), *N*,*N*′-dicyclohexylcarbodiimide (8.08 g, 6 equiv), and 4-dimethylaminopyridine(0.397 g, 0.5 equiv) were dissolved in 30 mL of tetrahydrofuran (THF). The mixture was degassed by a nitrogen flow, and the reaction occurred over 48 h under reflux at 75 °C. After quenching the reaction, the medium was filtered and solvent was removed. The products were solubilized in 40 mL of hexane and washed 5 times with 5 mL of 50:50 acetonitrile–methanol. The hexane phase was evaporated under vacuum, and the product was characterized via ^1^H NMR in deuterated chloroform. Thereafter, the yellow oily product was mixed with azobisisobutyronitrile (0.1968 g, 0.2 equiv) and (3-mercaptopropyl)trimethoxysilane (2.59 g, 2.2 equiv) and dissolved in THF (30 mL). After degassing with nitrogen, thiol-ene reaction occurred over 24 h under reflux at 80 °C. The medium was carefully removed to avoid any contact with ambient air. A small part of the product was then collected, purified with process previously reported for the Steglich esterification, and analyzed by ^1^H NMR.

#### 2.2.2. Functionalization of PEG_45_ with Short Alkyl Chain (C_8_) and Trimethoxysilane End Groups (**45-Alk8-TMS**)

**Figure 1:**
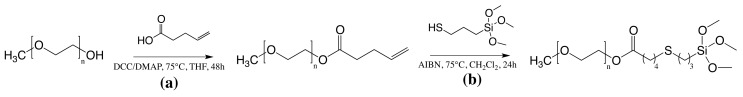
Synthesis of poly(ethylene glycol) (PEG) with short alkyl chain (C_8_) and trimethoxysilane end group. (**a**) Steglich esterification. (**b**) Thiol-ene coupling.

**45-Alk8-TMS** was prepared using Steglich esterification and thiol-ene (Figure 1) of PEG_45_ (1 g, 1 equiv), pentenoic acid (0.1 g, 2 equiv), *N*,*N*′-dicyclohexylcarbodiimide (0.206 g, 2 equiv), and 4-dimethylaminopyridine (0.0305 g, 0.5 equiv) were dissolved in dichloromethane (5 mL). The mixture was degassed by a nitrogen flow and the reaction occurred for 48 h under reflux at 75 °C. After quenching the reaction, the medium was filtered, and the solvent was removed. Products were solubilized in low THF volume and precipitated in diethyl ether. The solid was gathered and dried under vacuum. The product was characterized by ^1^H NMR and thereafter (2 g, 1 equiv) was mixed with azobisisobutyronitrile (0.0656 g, 0.2 equiv), (3-mercaptopropyl)trimethoxysilane (0.216 g, 1.1 equiv) and finally dissolved in THF (5 mL). The medium was placed under nitrogen, and the thiol-ene reaction occurred over 24 h under reflux at 80 °C. Afterward, while avoiding any contact with ambient air, the solvent was removed under vacuum. A small part of the product was then collected and purified before the Steglich esterification process reported above, and finally analyzed via ^1^H NMR. 

#### 2.2.3. Functionalization of PEG_45_ with Alkyl Chain C_14_ and Trimethoxysilane End Groups (**45-Alk14-TMS**)

**45-Alk14-TMS** was prepared using the same procedure described above for **45-Alk8-TMS**. The products used for Steglich esterification were poly(ethylene glycol) 45 (1 g, 1 equiv), undecenoic acid (0.186 g, 2 equiv), *N*,*N*′-dicyclohexylcarbodiimide (0.206 g, 2 equiv), and 4-dimethylaminopyridine (0.0305 g, 0.5 equiv). The product obtained (2 g, 1 equiv) was mixed with azobisisobutyronitrile (0.658 g, 0.2 equiv) and (3-mercaptopropyl)trimethoxysilane (0.213 g, 1.1 equiv) and, after treatment, was analyzed by ^1^H NMR.

#### 2.2.4. Film Preparation

**Figure 2:**
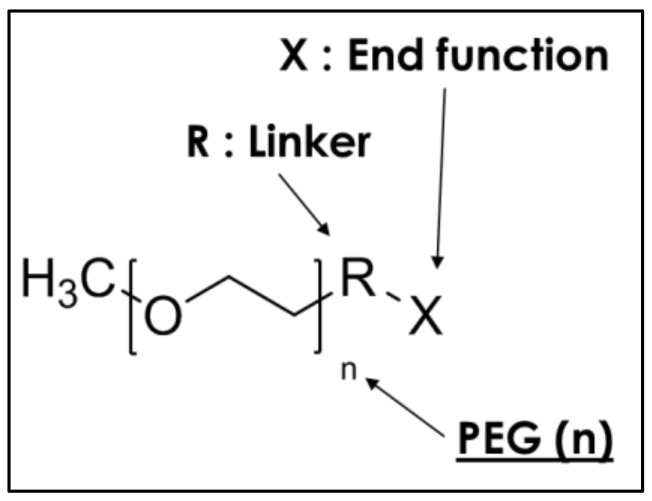
Structure of PEG-based additives.

The structure was composed of different number of ethylene oxide units (poly(ethylene glycol)) attached with a linker “R” to the polymer terminal function “X” (Figure 2). Details about the number of units, the structure of the linker, and the structure of the terminal function can be found in Table 1. The reference sample was composed of PDMS (13 or 28 units), to study the influence of the PDMS’ molecular weight, the ethanol (50 wt % of the PDMS), and the phosphoric acid (10 wt % of the PDMS). The PEG-based additives (10 wt % of the PDMS) were first mixed with ethanol, the PDMS bis(trimethoxysilane) end-groups were then placed into a small vial, and the phosphoric acid was added (10 wt % of the PDMS). After each addition, the reaction medium was homogenized for 2 min with a vortex. The product was coated on a polycarbonate (PC) sheet or a glass slide with a wet film thickness of 200 µm. Glass slides and PC sheets were cured at 20 °C for 48 h, and the samples were then stored in a drying oven at 30 °C. The film was removed from the PC sheets so that swelling test, soxhlet extraction, and scanning electron microscopy could proceed.

### 2.3. Characterizations

#### 2.3.1. Nuclear Magnetic Resonance

^1^H NMR spectra were obtained on a Spinsolve Benchtop NMR 60 MHz and proceeded in CDCl_3_. The esterification yield of PDMS was determined by the shift of protons close to the reactive function, 3.34–3.98 ppm (m, 2H, *CH_2_*OH). Esterification of PEG was confirmed by the apparition of allyl protons peak, 6.34–5.36 ppm (m, 2H, CH_2_*CH*CH_2_) and 5.34–4.62 ppm (m, 4H, *CH_2_*CHCH_2_), and protons peak close to the ester function, 3.96–4.43 ppm (m, 4H, *CH_2_*COOCH_2_). Thiol-ene coupling yield for all compounds was determined by the decrease of allyl protons peaks.

#### 2.3.2. Scanning Electron Microscopy

Coatings were observed with an electron microscope JEOL 6460 LV equipped with an Oxford Inca 300 X-Ray microanalysis. Samples were separated from the polycarbonate sheet and placed on the metallization support. The surface was then metallized with carbon to obtain a conductive material. Each sample was scanned, and a screenshot of the most representative global architecture was taken (magnification 100×). For all investigations, the beam energy was 20 kV.

#### 2.3.3. Soxhlet Extraction

The amount of un-cross-linked material in a film cured on a polycarbonate was determined by soxhlet extraction. The film was extracted with chloroform in a soxhlet device for 24 h. The weight loss was calculated as the weight difference between the extracted and unextracted weight divided by the unextracted weight.

#### 2.3.4. Contact Angle Measurement

Measurements were taken at room temperature with a contact angle system (Digidrop GBX, Dublin, Ireland) equipped with a syringe, a video camera, and an acquisition of angle measurements. The film was cured on the glass slide and then put in a drying oven at 30 °C for a week before contact measurements to have repeatable conditions. The contact angle of a 2 µL drop of water was measured at 0, 15, and 30 s after contact between the drop and the film surface. The indicated values are an average of 5–8 measurements taken on different areas of three different films.

## 3. Results

### 3.1. Synthesis 

#### 3.1.1. Synthesis of Dihydroxylalkyl Polydimethylsiloxane (PDMS_28_)

Dry product was analyzed by ^1^H NMR. The shift of protons from 3.34–3.98 to 3.76–4.40 ppm is due to the transformation of the hydroxyl group into an ester group (Figures S1). Its relative intensity compares to the one of the dimethyl peaks in the PDMS backbone to give a yield close to 100%. Moreover, the presence of peaks corresponding to allyl protons at 6.34–5.36 ppm and 5.34–4.62 ppm and their relative intensities of 2H and 4H, respectively, confirm the high yield obtained. Analysis of the ^1^H NMR spectrum after the thiol-ene reaction shows a decrease of the allyl protons peaks. The yield calculated from the relative intensities is 93%. Moreover, the presence of a methoxy proton peak (3.43–3.65 ppm) after purification confirms the end-group modification. (Figures S1 and S2).

#### 3.1.2. Poly(Ethylene Glycol) End-Group Modifications

^1^H NMR spectra of purified compounds obtained after the Steglich esterification show peaks corresponding to allyl protons. These peaks confirm the end-group modification for both linker types with different sizes (Figure S3 and S4).

As with the thiol-ene reaction of PDMS, the decrease of allyl proton peaks on spectra confirm the coupling between the PEG and the (3-mercaptopropyl)trimethoxysilane. Relative intensities indicate a yield of 74% for **45-Alk8-TMS** and 78% for **45-Alk14-TMS**.

### 3.2. Film Preparation

The solid additives **45-Alk14-TMS**, **45-Alk8-TMS**, and **45-PEG-OH** were solubilized with the addition of ethanol at 50 wt % of PDMS matrix weight. These PEG-based additives contain 45 ethylene oxide (EO) units, making their melting points higher than room temperature, which explains their solid state. They have to first be solubilized before their incorporation in the coatings for good dispersion [23]. The amount of matrix, solvent, catalyst, and additives for each film were kept consistent. This allows for samples to be compared under similar cross-linking conditions, with the only differing factor as the additive. Coatings obtained after 48 h in the oven are colorless, smooth, and without cracks.

### 3.3. Scanning Electron Microscopy

**Figure 3:**
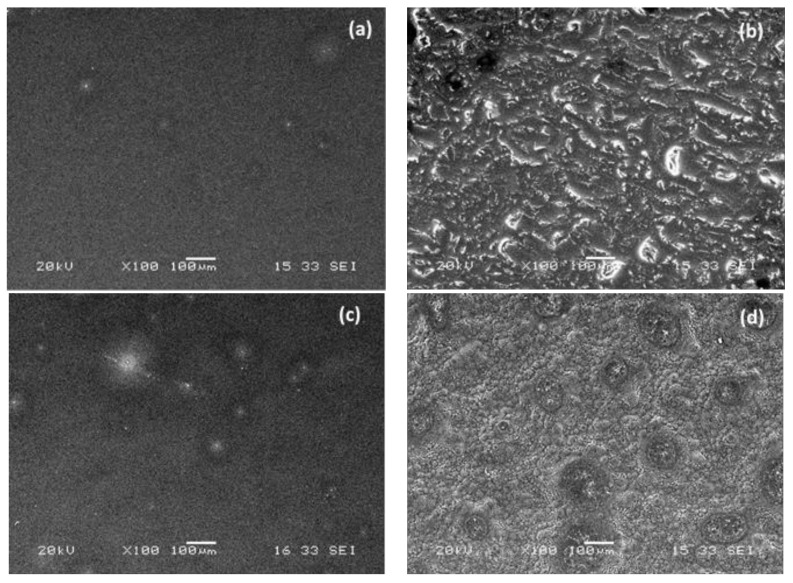
Scanning electron microscopy. Homogeneity of PDMS_13_-methoxy-ended cross-linked film with hydrophilic compounds incorporated (**a**) **6-TMS** (**b**) **45-OH** (**c**) **PDMS-TMS**, and (**d**) **45-Alk8-TMS.**

The PDMS reference presented a smooth surface indicating that the cross-linking system and the catalyst-solvent combination-proportion are efficient. The coatings prepared with **PDMS_28_** present the best results for film containing liquid hydrophilic additives as **PDMS-TMS_._** Nevertheless, their surfaces are less homogeneous, the linking of hydrophilic additives is less important and there is no surface accessibility compared to those with **PDMS_13_**. In addition, the surface presents a high amount of bubbles and drying defaults due to its higher cross-linking speed. The crosslinking and solvent-catalyst proportion are not suitable for **PDMS_28_** (Figure 3).

Surface homogeneity is essential for avoiding bio-adhesion: Dantas et al. [24] observed that reduction in the surface roughness was directly related to a decrease in bacterial adhesion. To have amphiphilic character over the whole surface, it is important to have a homogeneous material. The surface topography of **PDMS_13_**-based films is shown in Figure 3. There are two main types of surface films. The first is a smooth and homogeneous surface shown in Figure 3a,c for **6-TMS** and **PDMS-TMS**, respectively. The **PDMS reference**, **9-OH**, and **PDMS-SiOH** coatings exhibited similar surface properties. This smooth and homogeneous surface is characteristic of PDMS reference and four other coatings that contain only liquid hydrophilic additive with fewer EO repeat units. The additives **6-TMS** and **9-OH** contain 6 and 9 EO units, respectively. The **PDMS-SiOH** and **PDMS-TMS** contain 20–50 EO units per PDMS chain and should be solid according to Majumdar et al. [23]. However, the EO units are split into several low repeat PEG branches along the PDMS backbone, so the product remains in a liquid state even with an EO content superior to 13 units. These compounds are present in the liquid state and remain liquids after drying. Thus, film homogeneity should not be disrupted by samples with higher EO content adopting a solid state. Surface homogeneity seems to be impacted by the number of consecutive EO units in the PEG chain, as opposed to the total. The second typical profile, which presents with aggregation and a rough surface, is shown in Figure 3b,d for **45-OH** and **45-Alk8-TMS**, respectively. The **45-Alk14-TMS** sample was found to have the same kind of surface, but it is not shown here. This profile is thought to be due to solidification of the PEG. Indeed, PEG at 20 °C with 13 EO units or fewer (600 g·mol^−1^) are in a liquid state, and those above 13 units are present in a solid state [23]. These products have 45 EO units and are present in a solid state at 20 °C as expected. The heterogeneous surface and phase segregation is believed to be caused by the return of PEG to its solid state during the drying process. It has been noted that phase compatibilization was not improved despite the use of C_8_ and C_14_ linkers. The number of consecutive units of EO chain seems to be the only impacting factor.

Short linear PEG additives (where EO units < 13) or PEG-PDMS copolymers (where EO units < 13) are a good solution for obtaining homogeneous surfaces [23]. We conclude that it is also possible to use a copolymer with a pendant chain that allows for the incorporation of several EO units while keeping it in a liquid state and preserving the homogeneity of the surface.

### 3.4. Soxhlet Extraction

Chemical additive retention was tested on hydrophilic compounds with varying cross-linking function and linkers. This included evaluating PEG of different molecular weights to study the entanglement possibilities between the two compounds.

**Figure 4:**
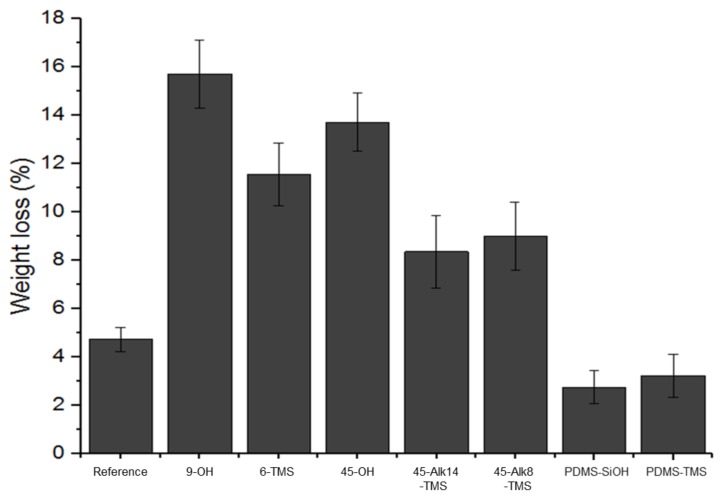
Uncross-linked materials and hydrophilic additives extracted from the film of **PDMS_13_**.

Leaching potential of the different hydrophilic compounds inside the **PDMS_13_** and **PDMS_28_** were tested. Figure 4 shows the weight loss of each coating. The PDMS reference had a higher weight loss (4.7%) when compared to a similar study by Grunlan et al. [25]. This could be due to the catalyst amount and the length of PDMS chain in the silicone sample that was used. Higher catalyst proportions accelerate cross-linking, decrease the chain mobility, and lead to structure immobilization. Short PDMS chains decrease the network flexibility and therefore the chain mobility, leading to a faster structure immobilization (freezing). This freeze process stops the reaction and hinders chain linking to the matrix. These chains will be thereafter extracted by solvent and contribute to the weight loss. 

In Figure 4, coatings containing **9-OH**, **6-TMS**, and **45-OH** presented high amounts of weight loss (15.7, 11.6, and 13.7%, respectively) consistent with the absence of linking. Differences in these samples were not significant based on their standard deviations. As mentioned, weight loss from the hydrophilic additives **9-OH** and **45-OH** are due to a lack of linking. In addition, increasing the number of EO units to 45 does not provide any noticeable entanglement effects between the PEG and PDMS network. The **6-TMS** structure is characterized by trimethoxysilane functionality, which induces cross-linking in the presence of water and H_3_PO_4_. However, the results suggest an absence of cross-linking with the PDMS. One advanced explanation is that the incompatibility between PEG and PDMS could lead to the gathering and collapsing of EO units around the cross-linking functional group. These phenomena would prevent the contact between the cross-linking function carried by the PEG and those present in the PDMS network, thus inhibiting the linking between these polymers.

Coatings containing the **45-Alk14-TMS** and **45-Alk8-TMS** additives showed similar behavior, with 8.4 and 9.0% weight loss. These compounds present reactive cross-linking functional groups like those of **6-TMS**. The linker between the EO units and the trimethoxysilane group may protect the cross-linking ability against the collapsing of EO and the PEG cluster formation. However, it could be ineffective in avoiding the gathering of EO, which also decreases the accessibility between PEG and PDMS functions. This gathering seems to be confirmed by the domains visible in SEM images. For these products, we believe that the PEG chains collapse but do not end up surrounding the cross-linking group. Thus, to obtain efficient linking, the TMS group, by using a linker and by compatibilizing the hydrophilic additives with the PDMS matrix, should remain protected from both PEG collapsing and gathering.

**PDMS_13_** coatings formulated with **PDMS-SiOH** and **PDMS-TMS** additives resulted in very low weight losses of 2.8 and 3.2%, respectively. This was unexpected, as these values were even lower than that of the PDMS reference (4.7%). This indicates that these additives can be efficiently linked to the network and improve the cross-linking of the network itself. One possible explanation is that the presence of EO units on a very fluid copolymer increases the compatibility between ethanol and PDMS, and facilitates chain mobility and overall cross-linking. Liquid copolymers might act as lubricants that facilitate the mobility and cross-linking of the PDMS chains. PDMS linkers to small PEG chains (EO units < 13) combined with a linking function yield good linking properties in the PDMS network. The coatings prepared with **PDMS_28_** showed similar trends, and, again, coatings prepared with **PDMS-SiOH** and **PDMS-TMS** additives showed the lowest weight loss. However, each coating prepared with **PDMS_28_** had larger amounts of weight loss compared to their respective **PDMS_13_** variants.

### 3.5. Contact Angle Measurements

**Figure 5:**
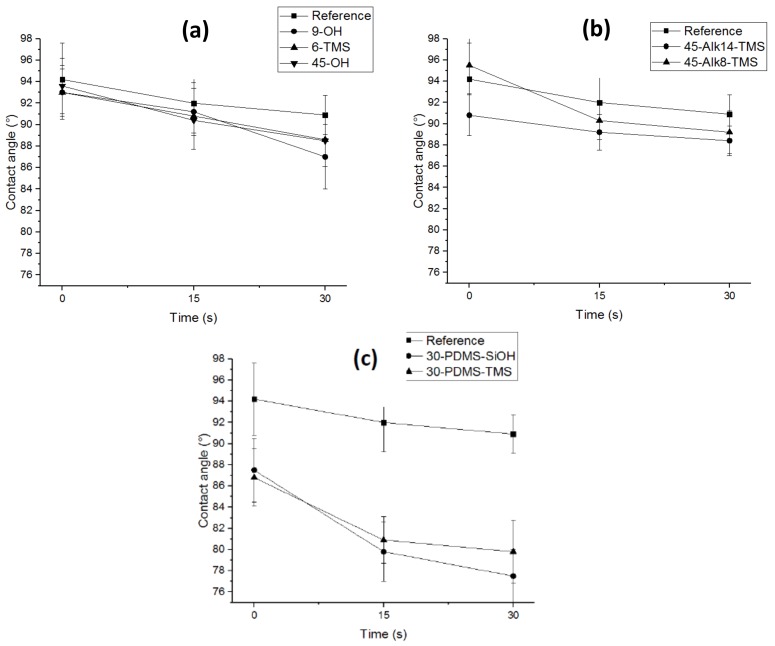
All hydrophilic additives were incorporated in the **PDMS_13_**-matrix-ended methoxy. (**a**) Water contact angle of hydrophilic additives with no linker. (**b**) Contact angle of hydrophilic additives with linker alkyl. (**c**) Contact angle of PEG additives with linker PDMS.

The amphiphilic character of the coating comes from the ability of the hydrophilic additives to migrate to the coating surface. This mobility could be provided by a high compatibilization between PDMS and the additives. The size of the additive should be a strong determinant as well. For non-cross-linkable additives, a low number of repeat units can provide high mobility. In contrast, linkable additives with a higher and sufficient number of repeat units will allow the attached chain to more easily access the surface. Contact angle results are shown in Figure 5 and demonstrate two main behaviors. The PDMS reference has an initial contact angle of 94° ± 2, followed by 91° ± 3 and 88° ± 3 at 15 and 30 s, respectively. Five other coatings presented similarly compared to the PDMS reference: **9-OH**, **6-TMS**, **45-OH**, **45-Alk8-TMS**, and **45-Alk14-TMS** (Figure 5a,b). Two things must be noted about these coatings: (1) The contact angle at *t* = 0 does not decrease, which means that there are probably no EO units at the surface. (2) The contact angles at *t* = 15 s and *t* = 30 s do not decrease significantly (with respect to PDMS reference), which could mean that the EO units cannot access the surface during this period of time. The most likely cause for the absence of EO units at the surface is that the hydrophilic glass slide may attract the PEG chains during the cross-linking. Furthermore, the lack of compatibility between these products and the PDMS matrix might restrain the chain mobility and hence the surface access.

Concerning the **45-Alk8-TMS** and **45-Alk14-TMS** coatings, the linking is slightly increased as demonstrated above and the surface access may be enhanced due to the length of the PEG chains. However, the 45 EO units may collapse and be entangled in the PDMS matrix, limiting their mobility and the surface accessibility. Equivalent systems with lower number of EO units have shown good surface access and decreased contact angles [15,23]. Shorter PEG chains (EO < 13) should be used with C_8_–C_14_ linkers and linking functionality to obtain efficient surface accessibility.

Coatings containing **PDMS-SiOH** and **PDMS-TMS** additives had lower initial contact angles followed by a noticeable decrease. The coatings prepared with **PDMS-SiOH** and **PDMS-TMS** additives seem thus to have EO units present on the surface, even at *t* = 0, demonstrating the ability of the hydrophilic additive to access the surface and increase the hydrophilicity. This may indicate that the hydrophilic additive is compatible and well dispersed within the PDMS matrix and does not accumulate particularly inside or at the bottom of the coatings. The decreasing contact angle over time indicates that the chains have sufficient mobility provided by the good compatibilization between these additives and the silicone matrix. The PEG chains collapsing does not seem to be an issue for these additives, as it decreases neither the mobility nor the surface accessibility. PDMS linkers increase the compatibility and the dispersion of the additive. The additive mobility after cross-linking seems to support this due to the combination of PDMS linkers and the small PEG chains, which do not cause detrimental entanglement.

All results presented above concern coatings prepared with **PDMS_13._** Compared to their **PDMS_13_** variants, coatings prepared with **PDMS_28_** generally had higher initial contact angles (98° ± 2.1) and presented less noticeable decreases of its contact angle over time. The coatings prepared with **PDMS_28_** presented less homogeneous surfaces, reduced linking ability, and less surface accessibility. This might be explained by the catalyst–solvent system, which may be suitable for **PDMS_13_** but inappropriate for **PDMS_28_**. In this study, to obtain comparable results, all conditions were fixed and are the same for each coating (the amount of solvent, the amount of catalyst, the type of catalyst, the amount of additive, and the time of cross-linking) (Table S1).

## 4. Discussion

According to the results, a combination of three elements is needed to achieve an amphiphilic non-leachable and homogeneous surface: the number of EO units (PEG molecular weight), the presence of linkable groups, and the nature of the linker used. The first element, EO units, is critical toward achieving a homogeneous surface and with an amphiphilic character. Where the number of EO units was greater than 13, two issues could be observed: (1) the solidification of PEG during drying resulted in a heterogeneous surface, and (2) the gathering and collapsing of EO units around the linking group limited the bonding. Even with cross-linking functionality and an alkyl linker, the PEG additives **45-Alk8-TMS** and **45-Alk14-TMS** did not bond significantly with the network. A longer or more compatible linker seems necessary and/or the use of a lower molecular weight PEG. Indeed, a lower molecular weight PEG chain allows for the additive to be in a liquid state at room temperature (20 °C) [23], which facilitates the blending and drying of the film. Furthermore, lower molecular weight PEG can better access the surface. According to the results of the **6-TMS** coating, the addition of a linking group seems necessary but does not achieve sufficient linking and surface accessibility. Our hypothesis is that the EO units collapse around the linking group, rendering it ineffective. In addition, the surface accessibility seems to require a good compatibilization. A linker must be added between the linking group and the EO units to improve compatibilization of the additive. The use of a PDMS linker provides a good compatibilization between both polymers. The addition of this linker appears to prevent collapse of EO units and enable cross-linking, as evidenced by the soxhlet results. The compatibilization also seems good (SEM) as well as the surface accessibility. Both the **PDMS-TMS** and **PDMS-SiOH** coatings presented qualities of good amphiphilic, non-leachable, and homogeneous additives. The main leverage for homogeneity seems to be the number of EO units in the PEG chain. For the accessibility, the nature of the linker is most important. For the linking, the size of the linker and its nature are critical. **PDMS-TMS** and **PDMS-SiOH** are good additive candidates with all the desired properties (homogeneity, accessibility, and linking).

## 5. Conclusions

In this study, the compatibilization of different PEG additives in a PDMS matrix was studied. This compatibilization is a critical parameter in achieving a formation of non-leaching coatings. Different properties such as linking ability of the additive in the matrix, surface accessibility, and coating homogeneity have been observed and discussed. It is suggested that the most important element is the presence of a reactive linking group that maintains the additive in the film. The availability of these groups depends on the presence of a linker that prevents the hindrance of the reactive chain end by EO groups. Another important element is the number of consecutive EO units. To obtain a homogeneous surface and promote surface accessibility, the additives should have a small number of EO units (under approximately 13 consecutive EO units) so that they remain liquid at room temperature and prevent disruption of the PDMS network. A third key factor is the compatibility provided by the linker. This compatibility contributes to the achievement of a homogeneous surface and can boost the surface accessibility of the EO units.

The best coatings were obtained with the copolymers **PDMS-SiOH** and **PDMS-TMS**, which contain a cross-linking group, a PDMS linker, and small pendant PEG chains. The linking group assures the linking to the PDMS network. The PDMS linker acts as a spacer to keep the linking active and as a compatibilizer due to its great affinity with the PDMS matrix. This kind of structure therefore has a high potential as an additive for non-leaching amphiphilic coatings.

## Figures and Tables

**Table 1:** Composition of hydrophilic additives of poly(dimethyl siloxane) (PDMS) coatings.

n: PEG	R: Linker	X: End Function	Samples	States of Additive
0	-		Reference	-
6	-	Trimethoxysilane	6-TMS	Liquid
9	-	Hydroxy	9-OH	Liquid
X	PDMS	Silanol	PDMS-SiOH	Liquid
X	PDMS	Trimethoxysilane	PDMS-TMS	Liquid
45	-	Hydroxy	45-OH	White solid
45	Alkyl C_8_	Trimethoxysilane	45-Alk8-TMS	Yellow solid
45	Alkyl C_14_	Trimethoxysilane	45-Alk14-TMS	Yellow solid

## Supplementary Materials

The supplementary materials are available online at http://www.mdpi.com/2073-4360/10/4/445/s1.
