# Supplementary material for: Non-Leachable Hydrophilic Additives for Amphiphilic Coatings
Source: Polymers (Basel). 2018 Apr 16;10(4):445. doi: 10.3390/polym10040445 (PMC6415241; doi:10.3390/polym10040445)
Supplement: Supplementary file 1 [file polymers-10-00445-s001.pdf]

*polymers*

# Supplementary Materials

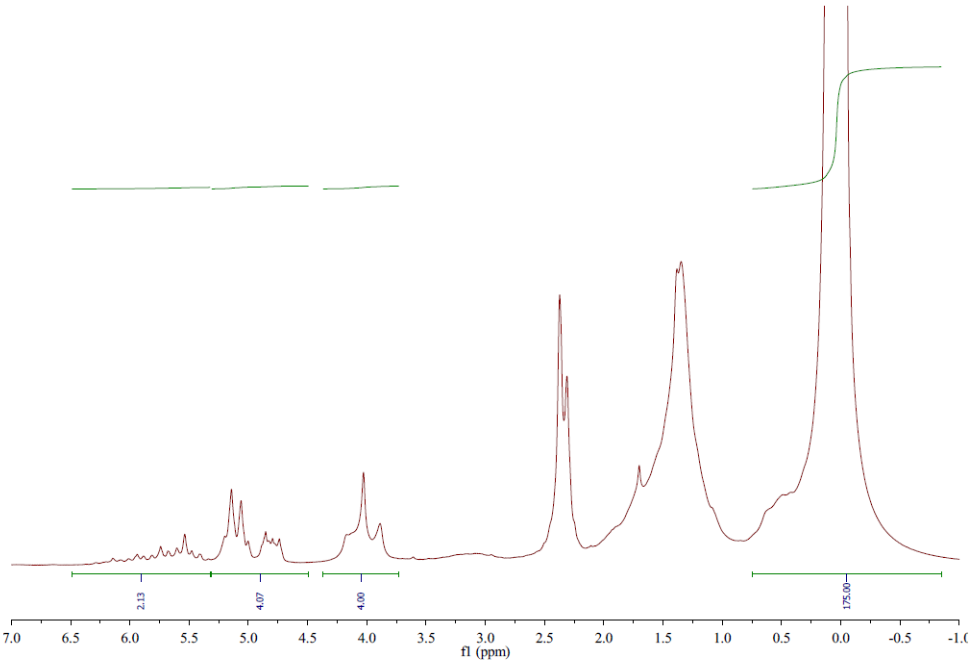


Figure S1 : NMR¹H of PDMS after Steglich esterification and purification.

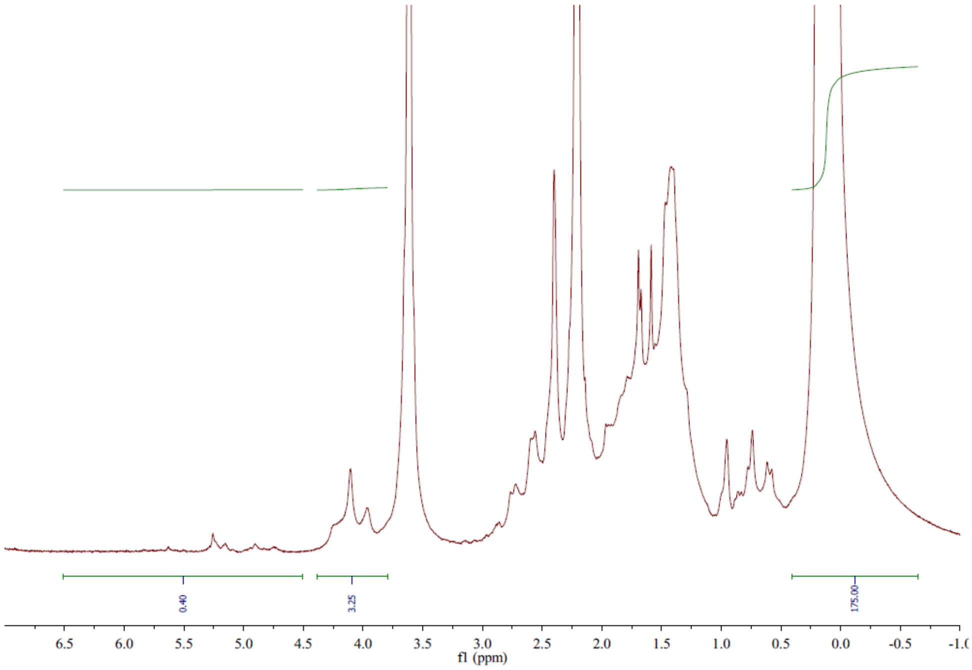


Figure S2 : NMR¹H of PDMS after thiol-ene reaction and purification.

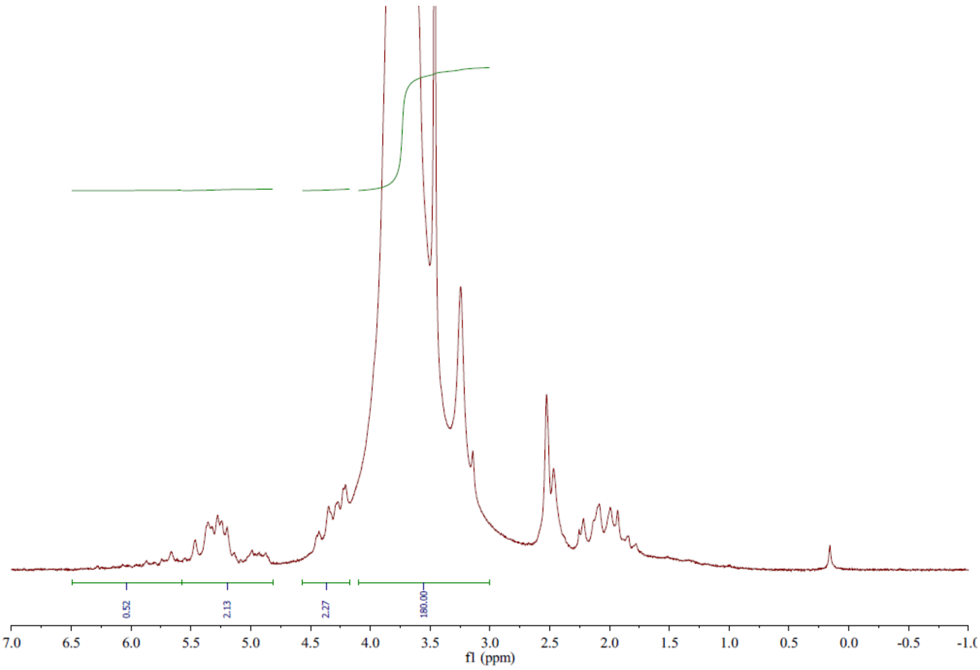


**F**igure S3 : NMR¹H of PEG (45-Alk8-TMS) after Steglich reaction and purification.

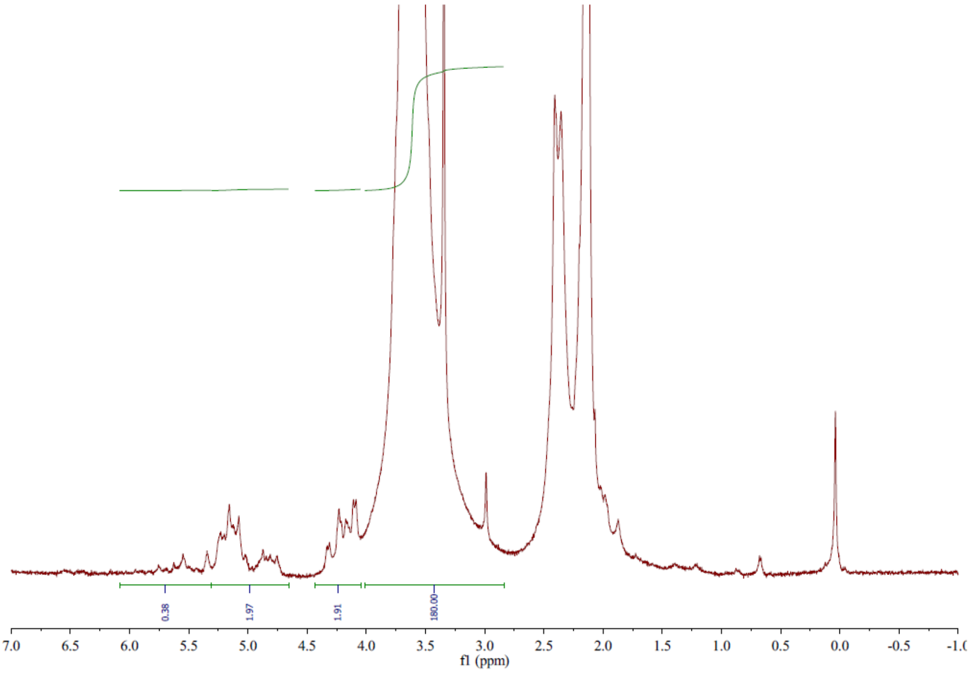


**F**igure S4 : NMR¹H of PEG (45-Alk14-TMS) after Steglich reaction and purification.

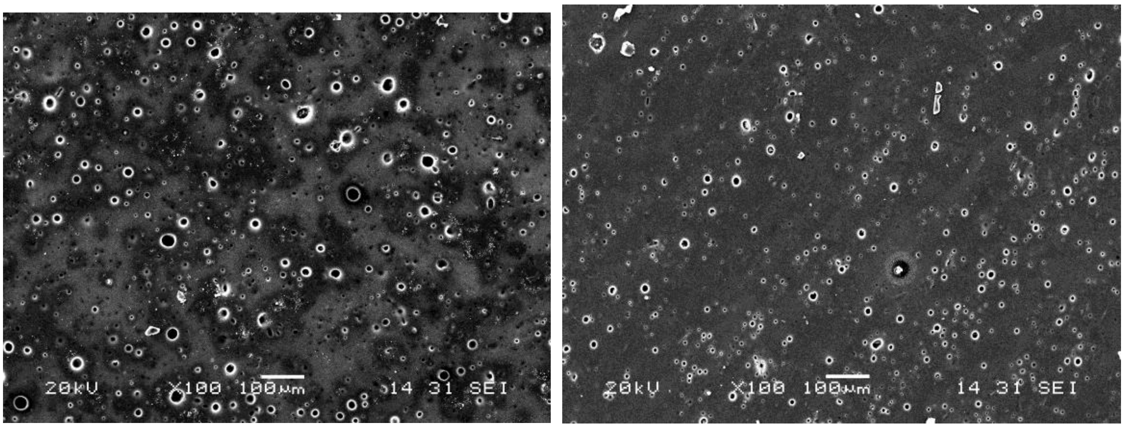


**F**igure S5 : Scanning Electron Microscopy. Homogeneity of $PDMS_{28}$ methoxy ended cross-linked film with hydrophilic compounds incorporated (**a**) ***45-OH*** (**b**) ***PDMS-SiOH.***

**T**able S1: Formulations of different coatings. Each sample is named as the PEG additives used for their formulation. Weight percentage are function of PDMS matrix weight**.**

| **Samples** | **Solvent (Ethanol)** | **Catalyst ($H_3PO_4$)** | **PEG additives** | **Time of cross linking** |
|---|---|---|---|---|
| Reference<br>6-TMS<br>9-OH<br>PDMS-SiOH<br>PDMS-TMS<br>45-OH<br>45-Alk8-TMS<br>45-Alk14-TMS | 50wt% | 1wt% | 10wt% | 24h at 20°C<br>then<br>48h at 30°C |
